# Tattoo Skin Disease in Cetacea: A Review, with New Cases for the Northeast Pacific

**DOI:** 10.3390/ani12243581

**Published:** 2022-12-18

**Authors:** Marie-Françoise Van Bressem, Koen Van Waerebeek, Pádraig J. Duignan

**Affiliations:** 1 Cetacean Conservation Medicine Group, Peruvian Centre for Cetacean Research, Museo de Delfines, Lima 20, Peru; 2 ProDelphinus, Miraflores, Lima 18, Peru; 3 The Marine Mammal Center, 2000 Bunker Road, Sausalito, CA 94965, USA

**Keywords:** disease, poxviruses, cetaceans, epidemiology, health, immunity

## Abstract

**Simple Summary:**

We review the literature on tattoo skin disease (TSD), a poxviral dermatopathy of cetaceans, and provide new insights. In addition, new necropsy reports for fifty-five harbour porpoises (*Phocoena phocoena*), twenty-two Delphinidae and four Kogiidae stranded in northern California in 2018–2021 were examined for TSD lesions. The disease occurs worldwide, in at least 21 cetacean species, with variable prevalence. Cetacean poxvirus (CePV)-1 and -2 were recovered from seven odontocetes and two mysticetes in the Americas, Europe and Hong Kong. Strains from Delphinidae are closely related. Among Phocoenidae, poxviruses were obtained only from harbour porpoises around the British Isles. In healthy odontocetes, an immune response develops over time, with young calves protected by maternal immunity. Salinity and sea surface temperature do not seem to influence TSD prevalence in free-ranging cetaceans. High concentrations of immunotoxic halogenated organochlorines may cause more severe disease. Off California, Delphinidae were less often (26.3%) affected by TSD than harbour porpoises (43.6%). Male porpoises were significantly more prone (58.1%) to show clinical disease than females (25.0%). Among males, TSD affected a high proportion of juveniles and subadults.

**Abstract:**

Tattoo skin disease (TSD) is a poxviral dermatopathy diagnosed in cetaceans. We review the literature on TSD aetiology, clinical characteristics, pathology and epidemiology and evaluate immune responses against the virus. In addition, necropsy reports for fifty-five harbour porpoises (*Phocoena phocoena*), twenty-two Delphinidae and four Kogiidae stranded in northern California in 2018–2021 were checked for diagnostic tattoo lesions. TSD occurs in the Mediterranean, North and Barents Seas, as well as in the Atlantic, eastern Pacific and Indian Oceans in at least 21 cetacean species, with varying prevalence. Two cetacean poxvirus (CePV) clades are recognised: CePV-1 in odontocetes and CePV-2 in mysticetes. CePV-1 isolates were recovered from six Delphinidae and one Phocoenidae in the Americas, Europe and Hong Kong. Strains from Delphinidae are closely related. Among Phocoenidae, poxviruses were sampled only in harbour porpoises around the British Isles. CePV-2 isolates were obtained from southern right whales (*Eubalaena australis*) and a bowhead whale (*Balaena mysticetus*). In healthy animals, an immune response develops over time, with young calves protected by maternal immunity. Salinity and sea surface temperature do not seem to influence TSD prevalence in free-ranging cetaceans. High concentrations of immunotoxic halogenated organochlorines may cause a more severe clinical disease. Substitution and loss of genes involved in anti-viral immunity may favour CePV entry, replication and persistence in the epidermis. Off California, Delphinidae were less often (26.3%) affected by TSD than harbour porpoises (43.6%). Male porpoises were significantly more prone (58.1%) to show clinical disease than females (25%). Among males, TSD affected a high proportion of juveniles and subadults. TSD was not detected in the Kogiidae.

## 1. Introduction

**Figure 1:**
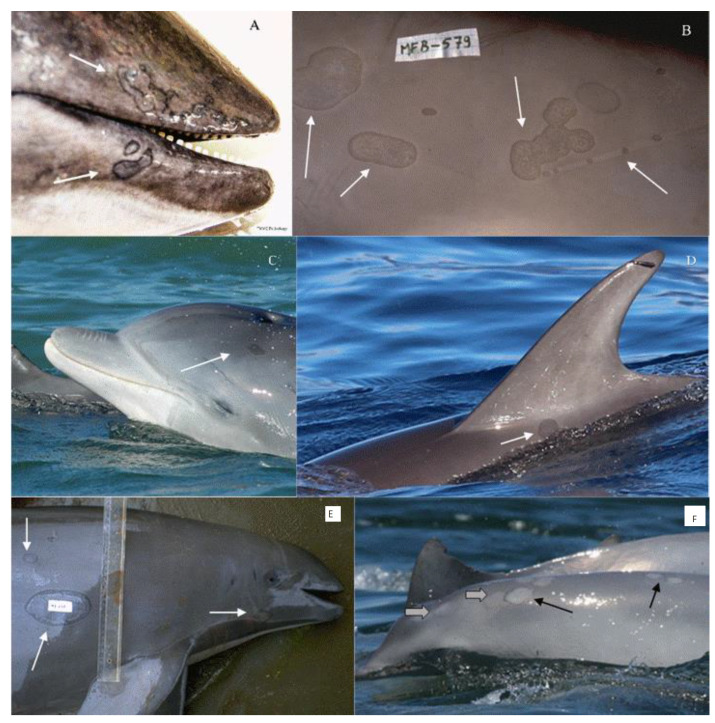
Tattoo skin lesions in (**A**) a harbour porpoise (*Phocoena phocoena*) from the north-central coast of California; (**B**) a dusky dolphin (*Lagenorhynchus obscurus*) caught off central Peru; (**C**) in a free-ranging common bottlenose dolphin (*Tursiops truncatus*) from the Pato Lagoon estuary, Brazil (courtesy of *P. Fruet*); (**D**) in a free-ranging Atlantic spotted dolphin (*Stenella frontalis*) from La Gomera, Canary Islands, Spain (courtesy of *F. Ritter*); (**E**) medium-sized and very large lesions in a Burmeister’s porpoise (*Phocoena spinipinnis*) from Peru; and (**F**) resolving lesions (black arrows) and tattoo remains (gray arrows) in a Guiana dolphin (*Sotalia guianensis*) from Sepetiba Bay, Brazil (courtesy of *L. Flach*).

Tattoo skin disease (TSD) is a poxviral, multifocal dermatopathy of Cetacea, macroscopically characterized by an irregular, variably extensive, gray or black stippling of the skin (Figure 1A–F) [1,2]. The perimeter of each active ‘tattoo’ is often hyperpigmented (Figure 1A,B) [1,2,3]. Reports worldwide are on the rise since its first description in common bottlenose dolphins (*Tursiops truncatus*), short-beaked common dolphins (*Delphinus delphis*) and an Atlantic white-sided dolphin (*Lagenorhynchus acutus*) in the 1970s [1,4,5]. 

Though its systemic impact is unclear, TSD is rarely lethal. However, it may affect individuals for months, causing large, circular, oval or irregular cutaneous lesions that expand centrifugally and may coalesce (Figure 1B,E). Electron microscopy and molecular studies have repeatedly confirmed the presence of poxviruses in acanthocytes of the affected epidermis as well as the specificity and sensitivity of TSD visual assessments, allowing a further insight into skin diseases of free-ranging cetaceans [1,2,6,7,8,9,10]. Here we review published information on TSD, provide an overview of its aetiology, its gross and histological characteristics as well as its epidemiology, and discuss further research imperatives. We also provide new data on TSD prevalence in free-ranging odontocetes stranded along the north central coast of California in 2018–2021 and examined at the Marine Mammal Centre in Sausalito.

## 2. Aetiology

**Figure 2:**
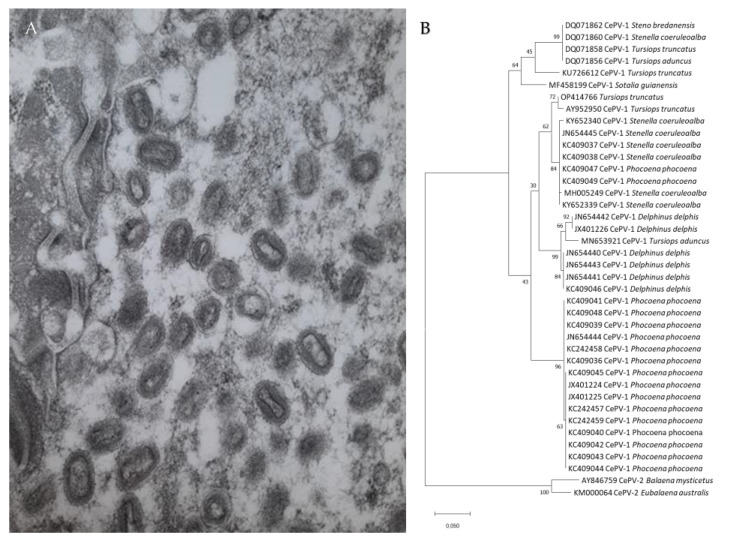
(**A**) Poxvirus particles in skin lesions of Burmeister’s porpoise (*Phocoena spinipinnis*) MFB-499 from central Peru (15,000 x magnification); (**B**) Phylogenetic tree among DNA polymerase nucleotide sequences of cetacean poxviruses available in GenBank “ adapted with permission from [10]. 2022, L. Luciani”.

TSD is caused by cetacean poxviruses (CePVs), members of the subfamily Chordopoxvirinae, tentatively classified into the genus *Cetaceanpoxvirus* which is awaiting acceptance by the International Committee on Taxonomy of Viruses (Figure 2A,B) [7,11,12]. Attempts to cultivate these double-stranded DNA viruses in vitro in cell cultures and on embryonated eggs have been unsuccessful to date [10,11].

A recent study provided the complete sequence of a cetacean poxvirus (CePV-TA) recovered from two tattoo skin lesions sampled in a captive Indo-Pacific bottlenose dolphin (*Tursiops aduncus*) [12]. The virus has one of the smallest poxvirus genomes (121,769 bp) and a low G + C content (28%). It comprises 120 open reading frames (ORFs), of which 80 encode proteins highly conserved among the subfamily Chordopoxvirinae. Five ORFs encode immune evasion proteins, including the apoptosis regulator ML11L-like proteins CePV-TA-4 and CePV-TA-117, the anti-apoptotic proteins CePV-TA-20 and CePV-TA-21 and the anti-inflammatory chemokine binding protein CePV-TA-113 [12]. All these immunomodulatory proteins are encoded in the host cell by the virus to inhibit the innate immune response [13]. CePV-TA is the only poxvirus that possesses two copies of the gene that encodes the E3L protein that, in vitro, inhibits activation of interferon-antiviral enzymes [12]. Phylogenetic analysis showed that CePV-TA proteins form a unique chordopoxvirus branch, basal to the clade comprising members of the genera *Centapoxvirus*, *Orthopoxviruses* and viruses of artiodactyls (*Cervidpoxvirus*, *Suipoxvirus*, *Capripoxvirus*, *Leporipoxvirus* and *Yatapoxvirus*) [12]. Genetic analysis of partial sequences of the DNA polymerase and DNA topoisomerase I genes indicate that the cetacean poxviruses of odontocetes and mysticetes belong to two different clades, namely CePV-1 and CePV-2, respectively (Figure 2B) [7,8,10,11,14]. CePV-1 includes strains recovered from Delphinidae (dolphins) and Phocoenidae (porpoises) from the North Atlantic Ocean, equatorial Atlantic Ocean, western South Atlantic Ocean, the eastern South Pacific and Arctic Ocean as well as from captive *T. aduncus* (Hong Kong) [7,9,10,11,12]. The Phocoenidae strains, all originating from harbour porpoises (*Phocoena phocoena*) of the eastern North Atlantic and North Sea, seem to form a separate lineage from the Delphinidae CePVs (Figure 2B) [7,8], but more data are necessary. Strains isolated from six species of Delphinidae are closely related irrespective of the host species or the ocean province where they occur [7,9,10]. For example, in the DNA polymerase region, the strain detected in a common bottlenose dolphin from the western South Atlantic Ocean (Brazil) shared the highest (95.5%) nucleotide identity with an isolate recovered in an Indo-Pacific bottlenose dolphin kept in captivity in Hong Kong and 98.8% amino acid similarity with a strain detected in a rough-toothed dolphin (*Steno bredanensis*) from the western North Atlantic (Florida) [9,11]. Thus, differences in TSD prevalence, severity and persistence are unlikely to be due to CePV genetic variation. 

Only two strains have been described from baleen whales, likely reflecting the difficulty of recognizing lesions on these species and obtaining samples for diagnostic testing. One strain was identified in skin opportunistically collected from a hunter-harvested bowhead whale (*Balaena mysticetus*) that did not have apparent lesions [11]. The second was from an adult female southern right whale (*Eubalaena australis*) that had raised, circular skin lesions when she stranded at Peninsula Valdes, Argentina [14]. 

## 3. Pathology

**Figure 3:**
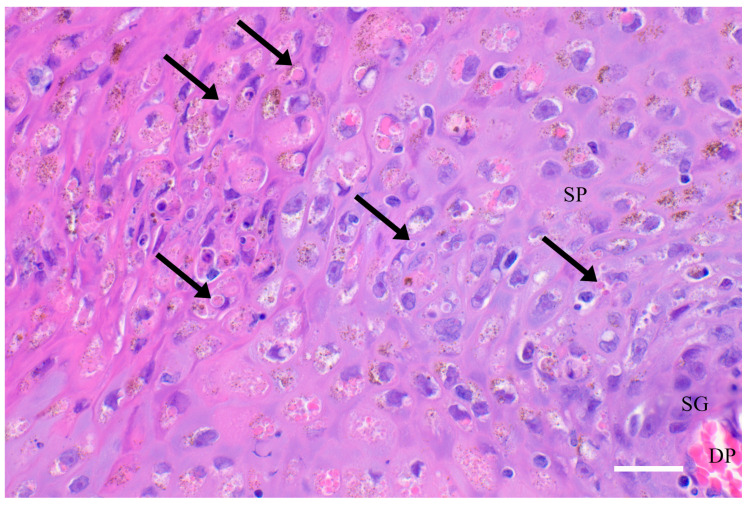
Histology and ultrastructure. Skin from a harbour porpoise (*Phocoena phocoena*) stranded along the coast of northern California with tattoo skin disease, showing a dermal papilla (DP) with capillaries, the stratum germinativum (SG) and part of the stratum spinosum (SP). Acanthocytes in the SP contain amphophilic cytoplasmic inclusion bodies (arrows). Hematoxylin & Eosin stain, 400x magnification, scale bar = 50μm.

At the histological level, there is focal cellular vacuolar degeneration in the stratum intermedium (aka stratum spinosum) and compaction of adjacent cells (Figure 3). The overlying stratum externum (aka corneum) is increased in depth by the proliferation of flattened cells extending into this layer. Viral replication is evidenced on light microscopy by variably sized eosinophilic cytoplasmic inclusion bodies, visible on transmission electron microscopy as enveloped dumbbell-shaped virions in cells of the transitional zone between the swollen vacuolated stratum intermedium and the compacted periphery (Figure 3) [1,5,15]. Leukocytic infiltration is generally mild and limited to lymphoplasmacytic infiltration at the dermal-epidermal junction and around superficial dermal blood vessels. Occasionally, the inflammation also includes some macrophages, eosinophils and neutrophils. In more chronic lesions, there is focal pitting and disruption of the surface layer, allowing entry of bacteria, fungi, protozoa and other opportunists. In these, there can be focally extensive necrosis in the stratum intermedium and externum with more marked leukocytic infiltration [1,15].

Macroscopically, the characteristic hyperpigmented stippled pattern and concentric growth of tattoo skin lesions is pathognomonic for cetacean poxvirus infection (Figure 1A–F) and a reliable diagnosis of TSD can often be made based on visual inspection of high-resolution photographs, including from at-sea observation [6,9,15,16]. Pathogenesis is still poorly understood. However, the multifocal skin lesions are regularly observed superposed on tooth rakes and other superficial epidermal injuries, suggesting that these may favour viral entry (Figure 1B). Time series images indicated that ‘tattoos’ enlarge centrifugally and can reach a very large diameter (Figure 1B–E) [1,3,6,15]. These skin lesions eventually heal, lose pigmentation and convert into pale grey marks (Figure 1F) before disappearing completely [6,8,9,16]. 

The presence of generalized tattoos, their location on different areas of the integument and the detection of poxvirus antibodies in the sera of several dolphins and porpoises [17,18] suggest a systemic spread of the virus. However, molecular analysis of different tissues (including brain, lungs, lymph nodes and kidneys) collected in a common bottlenose dolphin and a Guiana dolphin, both stranded along the Brazilian coast, detected poxviruses only in the tattoo skin lesions [9]. This implies that, if there is a viremia and infection of internal organs, the virus is rapidly cleared. In free-ranging *T. aduncus* from the relatively pristine waters of Shark Bay, Australia, TSD persisted for about 4.6 months [16], while in a community of *T. truncatus* inhabiting the contaminated Sado Estuary, Portugal, the symptomatic period varied between 3 and 45.5 months [19]. In a Guiana dolphin stranded in Rio de Janeiro, CePV genome could be detected by PCR one year after the first observation of TSD during a visual health assessment at sea [9]. The ability of CePV to persist in the skin for long periods may be due to several factors. A feature of cetacean epidermal cell kinetics is a slow turnover rate, with a transit time of 73 days calculated for bottlenose dolphins [20]. The longer retention of infected cells could allow for continued transmission within the epidermis over months. Other factors could be the relative lack of immune surveillance in the epidermis and/or the presence of at least five genes predicted to encode immune evasion proteins in the viral genome [12]. Immune fitness may also play a role and explain differences in severity and persistence between cetacean populations and communities. TSD is generally not a life-threatening disease either for free-ranging dolphins or those in captivity [1,6]. In a long-term study of free-ranging Indo Pacific bottlenose dolphins (*T. aduncus*) in Shark Bay, Western Australia, TSD occurrence was not significantly associated with survival to age three [16]. However, there is one report of a captive dolphin (species not mentioned but likely *Tursiops* sp.) that died in captivity from severe disseminated lesions [4]. 

## 4. Diagnosis

The stippled pattern and concentric growth of tattoo skin lesions is characteristic of the disease in cetaceans. Transmission electron microscopy has been used to identify poxvirus particles in tattoo skin samples (Figure 2A). Polymerase chain reaction (PCR) and sequencing of highly conserved regions within the poxvirus DNA polymerase and DNA topoisomerase I genes have been useful to identify CePVs in several species of odontocetes and mysticetes [7,8,11,14]. A recently developed pan-poxvirus PCR offers a new alternative to quickly detect these viruses, even in samples that were preserved in glutaraldehyde and DMSO for 30 years [10]. A non-invasive skin sampling device using sloughed skin sampled with cytology cell samplers proposes an alternative to the more intrusive biopsy sampling method in live dolphins [21].

## 5. Epidemiology

### 5.1. General Epidemiological Characteristics

Since its first description in the 1970s, TSD has been reported worldwide in 17 odontocetes and four mysticetes, from cold and warmer waters (Table 1). In free-ranging odontocetes, TSD prevalence varied widely between species and populations but generally showed similar epidemiological features. Thus, with the exception of Burmeister’s porpoises (*Phocoena spinipinnis*) from Peru, prevalence levels were similar in males and females [6,16]. TSD prevalence varied with age, peaking in juveniles compared to calves, likely because of the gradual loss of maternal immunity and increased contacts with infected pod members [16,22]. Prevalence generally decreases in mature animals, presumably following the development of acquired immunity [6,16,22].

In free-ranging *T. aduncus* from Shark Bay, the average age of an individual with tattoo skin lesions was 26.6 months and prevalence of the disease in adults was very low (0.33%, N = 303, [16]). Analysis of social networks and disease data from a longitudinal study in this *T. aduncus* community indicated that association with TSD-positive individuals increased the risk of contracting the disease [34]. Virus persistence in the cutaneous lesions, gregariousness and high levels of contacts between individuals likely ensure continuous infection and endemism in large and small cetacean communities [6,15,16,22,34]. 

### 5.2. Epidemiology of Tattoo Skin Disease in Odontocetes from the North Pacific

The prevalence of TSD in coastal odontocetes from the eastern North Pacific was data deficient. For this review, we surveyed cases necropsied at the Marine Mammal Center that included findings on fifty-five harbour porpoises, six short-beaked common dolphins, five Pacific white-sided dolphins (*Lagenorhynchus obliquidens*), five striped dolphins (*Stenella coeruleoalba*), three Risso’s dolphins (*Grampus griseus*), two northern right whale dolphins (*Lissodelphis borealis*), one common bottlenose dolphin and four pygmy sperm whales (*Kogia breviceps*) stranded along the north-central coast of California from January 2018 to December 2021. All carcasses were fresh and with intact epidermis (code 2 state of preservation) and all surfaces of the carcass were photographed prior to necropsy. The necropsy reports were reviewed for mention of tattoo skin disease or ‘tattoo lesions’ for this study. One of the authors (PJD) also reviewed all necropsy photographs. Additionally, histopathology was carried out on tissues from harbour porpoises (n = 24), Delphinidae (n = 17) and Kogiidae (n = 4) and characteristic acanthocyte vacuolation and intracytoplasmic inclusion bodies were present in the skin of four porpoises (Figure 2). Tattoo skin lesions were not detected either by gross or histologic examination in the pygmy sperm whales, northern right whale dolphins or the common bottlenose dolphin.

In the porpoises, tattoo lesions were most often observed on the head, but other body areas (trunk, flipper, peduncle) were also affected. However, for one juvenile male porpoise that was stranded in very poor body condition with severe nephrolithiasis and parasitic pneumonia, generalized skin lesions were spread over the body. Presumably, this individual was severely immunocompromised. TSD prevalence was 43.6% in this case series and twice as high in males (58.1%, n = 31) than in females (25%, n = 24) (Z score = 2.45; *p* = 0.0071; one-tailed), but the proportion of individuals per age class was not similar (Table 2), preventing further analysis. Prevalence was the highest in juvenile (81.8%, n = 11) and subadult (72.7%, n = 11) males, with no difference between them (Z = 0.509; *p* = 0.61) (Table 2). On the whole, this epidemiological pattern resembles the one observed in *P. spinipinnis* net-entangled off Peru (22), but differs from the pattern seen in healthy harbour porpoises from UK waters (6). The reasons for the higher infection prevalence in porpoises in the eastern North Pacific are unknown but may be related to stressors associated with residence in and adjacent to San Francisco, the second busiest shipping and boating harbour on the US west coast, habitats with high levels of contaminants, other human interactions such as with fisheries and inter-species aggression from common bottlenose dolphins [35,36,37,38]. The higher prevalence in males may reflect their accumulation of immunosuppressive lipophilic contaminants throughout life and the depuration of females through the transfer of their contaminant loads to their calves [39,40].

As their numbers by species were low, common dolphins, Pacific white-sided dolphins, striped dolphins and Risso’s dolphins were grouped into a ‘Delphinidae’ category. TSD prevalence was lower among them (26.3%, n = 19) than in harbour porpoises (43.6%), but not significantly so (Z = 1.33; *p* = 0.184). There was no evidence of variation by sex, but numbers were small (Table 2). With the exception of a female *G. griseus* calf (176 cm), all positives were juvenile and subadults. One male striped dolphin with numerous tattoo skin lesions had chronic arthritis and meningitis associated with *Brucella ceti* infection suggestive of a compromised immune response. 

### 5.3. Role of Stress and Health Status

Impaired immunity, poor health and stress may favour viral infection, persistence and recurrence, as described in *T. truncatus* [41,42]. Thus, the classical TSD epidemiological pattern observed in free-ranging, healthy odontocetes was altered in dolphins and porpoises from the British Isles, which were determined to have a lower health status, in which skin lesions were more prevalent in adults than in immature individuals [6]. Similarly, higher than expected TSD prevalence was reported in juvenile as well as in adult free-ranging *L. obscurus* and *P. spinipinnis* from Peru, with samples collected at a time when thousands were killed in direct and indirect fisheries [6,43]. For captive dolphins, Geraci et al. (1979) [1] observed that disease severity and recurrence was linked to general health in a *T. truncatus*, with TSD appearing and regressing concomitantly with episodes of bleeding gastric ulcers. In another study, visual assessment of TSD in 257 *T. truncatus* held in 31 facilities in the Northern Hemisphere indicated that, unlike in free-ranging Delphinidae, TSD prevalence was significantly higher in males (31.5%) than in females (12.3%) and that it remained high in adult males, but not in females. This suggested that males were more vulnerable to TSD than females, possibly because of a higher susceptibility to captivity-related stress and differences in immune response [44]. Stress likely enhances TSD recurrence, persistence and severity in both free-ranging and captive individuals [1,16,17,19,44].

### 5.4. Influence of Environmental Factors

Environmental factors such as temperature and salinity have also been suggested to influence TSD occurrence, prevalence and persistence [27,45]. A recent study in 25 *Tursiops* spp. kept in captivity at three facilities in Las Vegas and Orlando, USA, showed that, in 16 dolphins, tattoo skin lesions, some very large, changed colour and eventually disappeared after water temperature was increased from 20.6–22.8 °C to 24.4–27 °C. The time to complete resolution varied among the dolphins, with a range of 3 to 26 weeks [45]. In the other nine dolphins, the lesions faded but did not disappear during the study period [45]. When temperature was lowered to its previous values in one of the facilities, the tattoos recurred after four weeks in all three dolphins kept there [45]. Although de novo infection is a possibility [45], it is also conceivable that the poxvirus infection was not cleared in these dolphins, but that a higher epidermal cell turnover rate linked to the higher water temperatures masked the clinical appearance of the lesions.

These observations on the occurrence of TSD and water temperature in captive dolphins may not apply for free-ranging animals. Thus, high TSD prevalence levels (>20%) have been recorded in waters with an average sea surface temperature (SST) varying between 12.1 °C and 25.8 °C (Table 1). For instance, Burdett-Hart et al. (2012) [27] reported a 42.6% TSD prevalence in 101 common bottlenose dolphins free-ranging in Sarasota Bay, Florida, with a mean temperature of 24.4 °C but found a lower prevalence (19.9%) in 171 individuals from the colder waters (21.1 °C) of Charleston, South Carolina [27]. Furthermore, Sacristan et al., 2018 [9] reported that tattoo skin lesions, some quite large, persisted for several weeks and even months in three free-ranging Guiana dolphins from the warm waters (mean SST = 23.7 °C) of Guanabara Bay, Brazil (Table 1). Altogether these data indicate that, although high temperatures may resolve tattoo skin lesions macroscopically, or at least induce subclinical disease in captive dolphins, it may not be a major determinant of poxvirus infection in free-ranging cetaceans. A photo-identification (PI) study in three *T. truncatus* communities from South Carolina, Georgia and Florida showed that salinity was not significantly associated with the occurrence of tattoo skin lesions [27]. Similarly, no clear patterns related to geography and host phylogeny were observed during a study that examined the prevalence of TSD in several cetacean species and populations in oceanic, coastal and estuarine environments [6]. However, to our knowledge, the disease has never been recorded in freshwater dolphins. The most boreal documented case of cetacean poxvirus infection is in a bowhead whale from the Beaufort Sea, taken in the vicinity of Kaktovik (70°7′55″ N, 143°37′26″ W), northern Alaska in 1998 ([11], C. Romero pers. commn. 4 September 2022). The southernmost records of TSD were documented in Peale’s dolphins (*Lagenorhynchus australis*) and Chilean dolphins (*Cephalorhynchus eutropia*) at Guaitecas (43°53′ S 73°45′ W), Chilean Patagonia [6]. 

### 5.5. Impact of Immunotoxic Contaminants

Finally, chemical pollutants such as halogenated organic compounds (HOCs) that include polychlorinated biphenyls (PCBs), DDT and its derivatives, known to have immunomodulatory properties, may be present in the blubber of coastal cetaceans at high concentrations [46,47,48]. Indeed, the composition of contaminants may vary considerably between communities of the same species resident in different ocean provinces potentially complicating the clinical expression of similar diseases like TSD [49]. For *T. truncatus*, high blubber burdens of HOCs are associated with increased susceptibility to lobomycosis (lacaziosis), a fungal disease caused by *Paracoccidioides ceti* [41,50] and may also increase susceptibility to TSD. High levels of PCBs and dichlorodiphenyltrichloroethane were detected in the blubber and other tissues of two striped dolphins with TSD lesions in the Mediterranean Sea. One dolphin had very large (200 mm) lesions, suggesting that these contaminants may have played a role in the course and severity of the disease [30]. Similarly, harbour porpoises around the British Isles that were determined to be in a poor state of health had high levels of PCBs [39,51] as well as high TSD prevalence and numerous, sometimes very large, tattoos [6]. However, further research is needed to investigate the role of immune modulators and disease incidence and severity.

### 5.6. Tattoo Skin Disease in Baleen Whales

**Figure 4:**
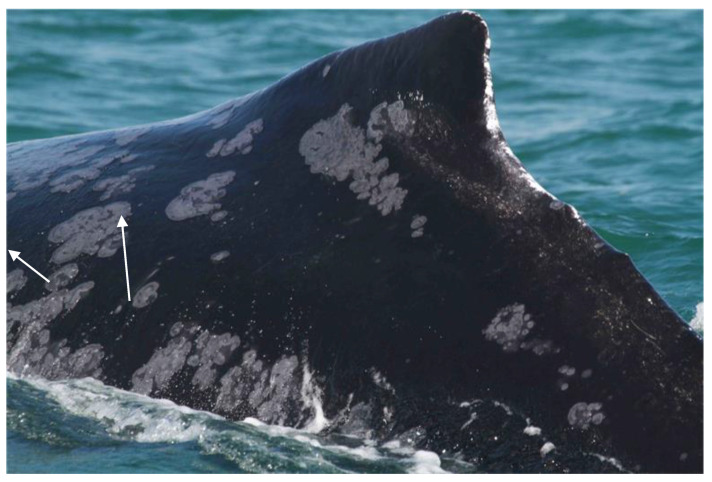
Numerous, small to large tattoo-like skin lesions (arrows) on the back and flank of a humpback whale (*Megaptera novaeangliae*) from Oman (Photo: copyright Environmental Society of Oman).

Although CePV-2 has only been identified in skin tissue from two mysticete species, TSD and TSD-like diseases have also been described in the North Atlantic right whale (*Eubalaena glacialis*) and the endangered, non-migratory Arabian Sea humpback whale (*Megaptera novaeangliae*, ASHW) population off Oman [33,52]. In 19 ASHW individuals, disease prevalence significantly increased in the period 2000–2018, from 27.6% in 2000–2011 to 51.7% in 2012–2018 [33]. Lesion size ranged from small to very large (Figure 4), covering over 50% of the visible body surface in some whales. The disease persisted for years in 10 whales with sufficient resight history [33,52]. This small population is threatened by entanglements in fishing gear and ship strikes, has low genetic diversity and a very low reproductive rate [33,53,54]. 

## 6. Immune Response to CePVs

Recent data indicated that odontocetes have lost the two Mixomavirus genes (Mx) that encode proteins defending mammals against a broad range of RNA and DNA viruses, by blocking early steps of the viral replication cycle [55,56]. In addition, the presence of cetacean-specific amino acid substitutions in interferon IL-6 and IL-27, as well as in the signal transducer and activator of transcription, may impair the mucosal immune response [57]. These losses of function may favour CePV entry, replication and persistence in the epidermis, as described for other viruses [55,57].

In mammals, the response to poxvirus infection is complex and powerful, involving innate as well as specific humoral and cellular immunity and varies according to the virus species and the host [58]. Several poxviruses, including members of the genera *Orthopoxvirus* and clade II poxviruses induce a long-lasting immunity preventing reinfection, while others like parapoxviruses do not [58,59]. Poxvirus-specific antibodies, along with T cells, are critical to resolve infection and protect against re-infection [60]. While our knowledge of immunity against cetacean poxviruses is limited, epidemiological data indicate that infected cetaceans develop immunity against the virus, with a consistently lower prevalence of tattoo skin lesions in adults [6,16,22]. Furthermore, the presence of poxvirus-specific antibodies has been described in the sera of captive and free-ranging odontocetes. Thus, the regression of typical tattoos was concurrent with antibody conversion in two captive *T. truncatus* with long-lasting lesions [17]. Cowpoxvirus (genus *Orthopoxvirus*) neutralising antibodies were detected in the sera of a high proportion of Peruvian dolphins (75.6%, N = 41) and porpoises (82.4%, N = 17), with titres varying between 40 and 1600 [18]. 

## 7. Conclusions

Over the past 20 years, research on tattoo skin disease and cetacean poxviruses has greatly increased. Molecular analysis (sequencing) has shed light on the taxonomy and phylogeny of cetacean poxviruses, demonstrating separate clades in odontocetes (CePV-1) and mysticetes (CePV-2) [7,10,11,12,14]. The development of high-definition digital photography, social network data analysis and proliferation of odontocete photo ID field studies worldwide have enabled visual assessments that provide deeper insights into the disease’s broad species and geographic distribution as well as into its epidemiology and ecology [6,9,16,19,29,33,34,44]. Future studies should attempt to sequence poxviruses from Phocoenidae species other than the harbour porpoise as well as from cetacean families that have not been sampled for CePV to date. They should also examine the link between high levels of chemical contaminants and TSD prevalence and severity. The role of stress in the pathogenesis and epidemiology must be further evaluated. Screening for poxvirus serum antibodies as well as evaluation of the virus presence in the internal organs of TSD affected individuals would improve our knowledge of CePV pathogenesis. Finally, whole genome analysis would provide crucial information to understand the genomic basis of the immune response to CePV, as described for cetacean morbillivirus [61].

## Figures and Tables

**Table 1:** Occurrence and prevalence levels (Prev) of tattoo skin disease (TSD) in free-ranging (FR), by-caught (BC), hunted (H) and stranded (S) odontocetes and mysticetes worldwide. Mean sea surface temperature (SST) is provided [https://www.seatemperature.org/, accessed on 18 November 2022)]; NA = not available; () = number of positive individuals; Ref = references.

Scientific Name	Sample Type	TSD Prev (%)	Total Number	Ocean Province	Mean SST	Ref.
*Delphinus delphis*	BC	3.6	28	SE Pacific (Ecuador, central coast)	23.8°C	[6]
*Tursiops aduncus*	S	3.6	112	Indian Ocean (Australia, St Vincent Gulf)	17.7°C	[23]
*Tursiops truncatus*	FR	4.5	334	Gibraltar Strait (Spain)	19.1°C	[24]
*Tursiops truncatus*	FR	5	79	SE Pacific (Peru, Paracas Bay)	18.1°C	[6]
*Sotalia guianensis*	FR	5.3	206	SW Atlantic (Brazil, Sepetiba Bay)	23.8°C	[6]
*Delphinus delphis*	BC	5.6	18	NE Atlantic (British Isles)	11.8°C	[6]
*Sousa sahulensis*	FR	9.9	91	SE Pacific (Moreton Bay, Australia)	23.1°C	[25]
*Stenella coeruleoalba*	S	7.5	40	Mediterranean Sea (Spain, Valencian Community)	19.3°C	[6]
*Tursiops aduncus*	FR	7	100	SE Pacific (Moreton Bay, Australia)	23.1°C	[25]
*Phocoena phocoena*	BC	10	10	North Sea (British Isles)	10.8°C	[6]
*Phocoena phocoena*	S	10.9	46	NE Atlantic (British Isles)	11.8°C	[6]
*Cephalorhynchus h.* *hectori*	S	11.1	27	SW Pacific & Tasman Sea (New Zealand, South Island)	13.8°C	[6]
*Delphinus delphis*	S	11.1	9	NE Atlantic (British Isles)	11.8°C	[6]
*Tursiops truncatus*	S	12.5	8	Mediterranean Sea (Spain, Valencian Community)	19.3°C	[6]
*Phocoena phocoena*	S	13.8	29	North Sea (British Isles)	10.8°C	[6]
*Tursiops aduncus*	FR	13.9	36	Indian Ocean (Australia, Swan-Canning Riverpark)	20.8°C	[26]
*Delphinus delphis*	S	16.7	6	NE Pacific (USA, northern California)	12.4°C	[This paper]
*Cephalorhynchus eutropia*	FR	17.4	23	SE Pacific (Chile, Guaitecas Archipelago, Aysén Region)	12.1°C	[6]
*Tursiops aduncus*	FR	19.4	247	Indian Ocean (Australia, Shark Bay)	22.5°C	[16]
*Tursiops truncatus*	FR	19.9	171	NW Atlantic (USA, South Carolina)	21.1°C	[27]
*Tursiops truncatus*	FR	20	35	NE Atlantic (Portugal, Sado Estuary)	17°C	[19]
*Lagenorhynchus obliquidens*	S	20	5	NE Pacific (USA, northern California)	12.4°C	[This paper]
*Tursiops truncatus*	FR	21	189	NW Atlantic (USA, Georgia)	20.7°C	[27]
*Cephalorhynchus h. hectori*	BC	24.3	37	SW Pacific & Tasman Sea (New Zealand, South Island)	13.3°C	[6]
*Lagenorhynchus australis*	FR	27.6	29	SE Pacific (Chile, Guaitecas Archipelago, Aysén Region)	12.1°C	[6]
*Tursiops truncatus*	FR	27.9	43	NW Atlantic (USA, FL, Indian River Lagoon)	25.8°C	[28]
*Grampus griseus*	S	33.3	3	NE Pacific (USA, northern California)	12.4°C	[This paper]
*Lagenorhynchus obscurus*	BC	34.7	196	SE Pacific (Peru, central coast)	18°C	[22]
*Lagenorhynchus australis*	FR	39.1	115	SE Pacific (Chile, Añihué Reserve, Aysén Region)	12.1°C	[3]
*Stenella coeruleoalba*	S	40	5	NE Pacific (USA, northern California)	12.4°C	[This paper]
*Tursiops truncatus*	BC	41.6	12	SE Pacific (Peru, central coast)	18°C	[22]
*Tursiops truncatus*	FR	42.6	101	NW Atlantic (USA, FL, Sarasota Bay)	24.4°C	[27]
*Phocoena phocoena*	S	43.6	55	NE Pacific (USA, northern California)	12.4°C	[This paper]
*Delphinus capensis*	BC	61.1	54	SE Pacific (Peru, central coast)	18°C	[22]
*Phocoena spinipinnis*	BC	62.3	77	SE Pacific (Peru, central coast)	18°C	[22]
*Stenella frontalis*	FR	(3)	NA	Central east Atlantic (La Gomera, Canary Islands)	21.4°C	[29]
*Stenella frontalis*	S	(1)	NA	Central east Atlantic (Teneriffe, Canary Islands)	21.1°C	[21]
*Tursiops truncatus*	FR	(2)	NA	Central east Atlantic (La Gomera, Canary Islands)	21.4°C	[29]
*Tursiops truncatus*	S	(1)	NA	Central east Atlantic (Teneriffe, Canary Islands)	21.1°C	[21]
*Sotalia guianensis*	FR & S	(5)	NA	SW Atlantic (Brazil, Guanabara Bay)	23.7°C	[9]
*Hyperoodon ampullatus*	S	(1)	NA	North Sea (British Isles)	10.8°C	[6]
*Steno bredanensis*	FR	(2)	NA	Central east Atlantic (La Gomera, Canary Islands)	21.4°C	[29]
*Delphinus delphis*	FR	(1)	NA	Central east Atlantic (La Gomera, Canary Islands)	21.4°C	[29]
*Stenella coeruleoalba*	S	(2)	NA	Mediterranean Sea (Italy, Anzio and Tuscany)	19.2°C	[30]
*Tursiops truncatus*	FR	(1)	NA	NE Pacific (Santa Monica, California)	16.6°C	[31]
*Balaena mysticetus*	H	(1)	NA	Beaufort Sea, Arctic (Kaktovik, Alaska)	0°C	[11]
*Eubalaena australis*	S	(2)	NA	SE Atlantic (Chubut, Argentina)	13.3°C	[14]
*Eubalaena glacialis*	FR	(1)	NA	NE Atlantic (New England, USA)	10.7°C	[32]
*Megaptera novaeangliae*	FR	41	93	Arabian Sea (Oman)	27.3°C	[33]

**Table 2:** TSD prevalence levels (Prev) in Phocoenidae and Delphinidae stranded along the Californian coast in 2018–2021. Abbreviations are: Nt = total number; N pos = number of individual positives with tattoo skin disease, as determined by visual examination and, in four cases, also by histology.

	Females			Males		
	Nt	N pos	Prev %	Nt	N pos	Prev %
***Phocoena phocoena***						
calf	8	0	0	7	1	14.3
juvenile	3	1	33.3	11	9	81.8
subadult	5	3	60.0	11	8	72.7
adult	8	2	25.0	2	0	0
Total	24	6	25.0	31	18	58.1
**Delphinidae**						
calf	3	1	33.3	1	0	0
juvenile	2	1	50.0	5	1	20.0
subadult	0	0	-	3	2	66.7
adult	2	0	0	3	0	0
Total	7	2	28.6	12	3	25.0

## References

[1] Geraci J.R., Hicks B.D., St Aubin D.J. (1979). Dolphin pox: A skin disease of cetaceans. Can. J. Comp. Med..

[2] Van Bressem M.F., Van Waerebeek K., Reyes J.C., Dekegel D., Pastoret P.P. (1993). Evidence of Poxvirus in Dusky Dolphin (*Lagenorhynchus obscurus*) and Burmeister’s Porpoise (*Phocoena spinipinnis*) from Coastal Peru. J. Wildl. Dis..

[3] Sanino G.P., Van Bressem M.-F., Van Waerebeek K., Pozo N. (2014). Skin disorders of coastal dolphins at Añihue Reserve, Chilean Patagonia: A matter of concern. Bol. Mus. Nac. Hist. Nat. Chile.

[4] Sweeney J.C., Ridgway S.H. (1975). Common diseases of small cetaceans. J. Am. Vet. Med. Assoc..

[5] Flom J.O., Houk E.J. (1979). Morphologic evidence of poxvirus in ‘tattoo’ lesions from captive bottlenosed dolphins. J. Wildl. Dis..

[6] Van Bressem M.-F., Van Waerebeek K., Aznar F.J., Raga J.A., Jepson P.D., Duignan P., Deaville R., Flach L., Viddi F., Baker J.R. (2009). Epidemiological pattern of tattoo skin disease: A potential general health indicator for cetaceans. Dis. Aquat. Org..

[7] Blacklaws B.A., Gajda A.M., Tippelt S., Jepson P.D., Deaville R., Van Bressem M.-F., Pearce G.P. (2013). Molecular characterization of poxviruses associated with tattoo skin lesions in UK cetaceans. PLoS ONE.

[8] Barnett J., Dastjerdi A., Davison N., Deaville R., Everest D., Peake J., Finnegan C., Jepson P., Steinbach F. (2015). Identification of Novel Cetacean Poxviruses in Cetaceans Stranded in South West England. PLoS ONE.

[9] Sacristán C., Esperón F., Marigo J., Ewbank A.C., de Carvalho R.R., Groch K.R., de Castilho P.V., Sánchez-Sarmiento A.M., Costa-Silva S., Ferreira-Machado E. (2018). Molecular identification and microscopic characterization of poxvirus in a Guiana dolphin and a common bottlenose dolphin, Brazil. Dis. Aquat. Org..

[10] Luciani L., Piorkowski G., De Lamballerie X., Van Waerebeek K., Van Bressem M.-F. (2022). Detection of Cetacean Poxvirus in Peruvian Common Bottlenose Dolphins *(Tursiops truncatus*) Using a Pan-Poxvirus PCR. Viruses.

[11] Bracht A.J., Brudek R.L., Ewing R.Y., Manire C.A., Burek K.A., Rosa C., Beckmen K.B., Maruniak J.E., Romero C.H. (2006). Genetic identification of novel poxviruses of cetaceans and pinnipeds. Arch. Virol..

[12] Rodrigues T.C., Subramaniam K., Varsani A., McFadden G., Schaefer A.M., Bossart G.D., Romero C.H., Waltzek T.B. (2020). Genome characterization of cetaceanpox virus from a managed Indo-Pacific bottlenose dolphin (*Tursiops aduncus*). Virus Res..

[13] Lu Y., Zhang L. (2020). DNA-Sensing Antiviral Innate Immunity in Poxvirus Infection. Front. Immunol..

[14] Fiorito C., Palacios C., Golemba M., Bratanich A., Argüelles M., Fazio A., Bertellotti M., Lombardo D. (2015). Identification, molecular and phylogenetic analysis of poxvirus in skin lesions of southern right whale. Dis. Aquat. Org..

[15] Duignan P.J., Van Bressem M.F., Cortés-Hinojosa G., Kennedy-Stoskopf S., Dierauf L.A., Gulland F.M.D. (2018). Viruses of marine mammals. CRC Handbook of Marine Mammal Medicine: Health, Disease, and Rehabilitation.

[16] Powell S.N., Wallen M.M., Bansal S., Mann J. (2018). Epidemiological Investigation of Tattoo-Like Skin Lesions among Bottlenose Dolphins in Shark Bay, Australia. Sci. Total Environ..

[17] Smith A.W., Skilling D.E., Ridgway S.H., Fenner C.A. (1983). Regression of cetacean tattoo lesions concurrent with conversion of precipitin antibody against a poxvirus. J. Am. Vet. Med. Assoc..

[18] Van Bressem M.F., van Waerebeek K., Bennett M. (2006). Orthopoxvirus neutralising antibodies in small cetaceans from the Southeast Pacific. Lat. Am. J. Aquat. Mamm..

[19] Van Bressem M.-F., Gaspar R., Aznar J. (2003). Epidemiology of tattoo skin disease in bottlenose dolphins (*Tursiops truncatus*) from the Sado estuary, Portugal. Dis. Aquat. Org..

[20] Hicks B.D., St Aubin D.J., Geraci J.R., Brown W.R. (1985). Epidermal growth in the bottlenose dolphin, *Tursiops truncatus*. J. Investig. Dermatol..

[21] Segura-Göthlin S., Fernández A., Arbelo M., Felipe-Jiménez I., Colom-Rivero A., Almunia J., Sierra E. (2021). The Validation of a Non-Invasive Skin Sampling Device for Detecting Cetacean Poxvirus. Animals.

[22] Van Bressem M.F., van Waerebeek K. (1996). Epidemiology of Poxvirus in Small Cetaceans from the Eastern South Pacific. Mar. Mammal. Sci..

[23] Tomo I., Kemper C.M. (2022). Strandings in St Vincent Gulf Bioregion, South Australia: 12-Year Study Monitors Biology and Pathology of Cetaceans. Oceans.

[24] Jiménez-Torres C., Verborgh P., de Stephanis R., Gauffier P., Esteban R., Giménez J., Van Bressem M.-F. A visual health assessment of a resident community of bottlenose dolphins in the Strait of Gibraltar. Proceedings of the 27th European Cetacean Society Conference.

[25] Hawkins E.R., Gustavsson M., Pogson-Manning L., Pheloung H., Jaehnichen C. (2022). Prevalence of Skin Lesions and Injuries in Australian Humpback Dolphins (*Sousa sahulensis*) and Indo-Pacific Bottlenose Dolphins (*Tursiops aduncus*) in Moreton Bay, Queensland. Aquat. Mamm..

[26] Chabanne D., Harrison L.M., Holyoake C., Finn H., Stephens N., Bejder L. (2012). Final Report to the Swan River Trust for Project RSP10MUR Technical Report.

[27] Burdett-Hart L., Rotstein D.S., Wells R.S., Allen J., Barleycorn A., Balmer B.C., Lane S.M., Speakman T., Zolman E.S., Stolen M. (2012). Skin lesions on common bottlenose dolphins (*Tursiops truncatus*) from three sites in the Northwest Atlantic, USA. PLoS ONE.

[28] Bossart G.D., Schaefer A.M., McCulloch S., Goldstein J., Fair P.A., Reif J.S. (2015). Mucocutaneous lesions in free-ranging Atlantic bottlenose dolphins *Tursiops truncatus* from the southeastern USA. Dis. Aquat. Organ..

[29] Kautek G., Van Bressem M.-F., Ritter F. (2018). External Body Conditions in Cetaceans from La Gomera, Canary Islands, Spain. J. Mar. Anim. Ecol..

[30] Cocumelli C., Fichi G., Marsili L., Senese M., Cardeti G., Cersini A., Ricci E., Garibaldi F., Scholl F., Di Guardo G. (2018). Cetacean Poxvirus in Two Striped Dolphins (*Stenella coeruleoalba*) Stranded on the Tyrrhenian Coast of Italy: Histopathological, Ultrastructural, Biomolecular, and Ecotoxicological Findings. Front. Veter. Sci..

[31] Bearzi M., Rapoport S., Chau J., Saylan C. (2009). Skin lesions and physical deformities of coastal and offshore common bottlenose dolphins (*Tursiops truncatus*) in Santa Monica Bay and adjacent areas, California. Ambio.

[32] Hamilton P.K., Marx M.K. (2005). Skin lesions on North Atlantic right whales: Categories, prevalence and change in occurrence in the 1990s. Dis. Aquat. Org..

[33] Minton G., van Bressem M.-F., Willson A., Collins T., Al Harthi S., Sarrouf Willson M., Baldwin R., Leslie M.S., van Waerebeek K. (2022). Visual health assessment and evaluation of anthropogenic threats to Arabian Sea humpback whales in Oman. J. Cetacean Res. Manag..

[34] Powell S.N., Wallen M.M., Miketa M.L., Foroughirad V., Bansal S., Mann J. (2020). Sociality and tattoo skin disease among bottlenose dolphins in Shark Bay, Australia. Behav. Ecol..

[35] Cope S., Hines E., Bland R., Davis J.D., Tougher B., Zetterlind V. (2021). Multi-sensor integration for an assessment of underwater radiated noise from common vessels in San Francisco Bay. The J. Acoust. Soc. Am..

[36] Sutton R., Chen D., Sun J., Greig D.J., Wu Y. (2019). Characterization of brominated, chlorinated, and phosphate flame retardants in San Francisco Bay, an urban estuary. Sci. Total Environ..

[37] Wilkin S.M., Cordaro J., Gulland F., Wheeler E., Dunkin R., Sigler T., Casper D., Berman M., Flannery M., Fire S. (2012). An unusual mortality event of harbor porpoises (*Phocoena phocoena*) off central California: Increase in blunt trauma rather than an epizootic. Aquat. Mamm..

[38] Wu Y., Tan H., Sutton R., Chen D. (2017). From sediment to top predators: Broad exposure of polyhalogenated carbazoles in San Francisco Bay (USA). Environ. Sci. Technol..

[39] Jepson P.D., Bennett P.M., Deaville R., Allchin C.R., Baker J.R., Law R.J. (2005). Relationships between polychlorinated biphenyls and health status in harbor porpoises (*Phocoena phocoena*) stranded in the United Kingdom. Environ. Toxicol. Chem..

[40] Wells R.S., Tornero V., Borrell A., Aguilar A., Rowles T.K., Rhinehart H.L., Hofmann S., Jarman W.M., Hohn A.A., Sweeney J.C. (2005). Integrating life-history and reproductive success data to examine potential relationships with organochlorine compounds for bottlenose dolphins (*Tursiops truncatus*) in Sarasota Bay, Florida. Sci. Total Environ..

[41] Reif J.S., Peden-Adams M.M., Romano T.A., Rice C.D., Fair P.A., Bossart G.D. (2009). Immune dysfunction in Atlantic bottlenose dolphins (*Tursiops truncatus*) with lobomycosis. Med. Mycol..

[42] Fair P.A., Schaefer A.M., Romano T., Bossart G.D., Lamb S.V., Reif J.S. (2014). Stress response of wild bottlenose dolphins (*Tursiops truncatus*) during capture-release health assessment studies. Gen. Comp. Endocrinol..

[43] Van Waerebeek K., van Bressem M.-F., Félix F., Alfaro J., García Godos A., Chavez L., Ontón K., Montes D., Bello R. (1997). Mortality of dolphins and porpoises in coastal fisheries off Peru and southern Ecuador in 1994. Biol. Cons..

[44] Van Bressem M.-F., van Waerebeek K., Duignan P.J. (2018). Epidemiology of tattoo skin disease in captive common bottlenose dolphins (*Tursiops truncatus*): Are males more vulnerable than females?. J. Appl. Anim. Welf. Sci..

[45] Croft L.A., Laughlin R., Manley M., Nollens H.H. (2020). Water temperature fluctuations as a key driver of cetacean pox (tattoo) lesions in bottlenose dolphins *Tursiops truncatus*. Dis. Aquat. Organ..

[46] Shaul N.J., Dodder N.G., Aluwihare L.I., Mackintosh S.A., Maruya K.A., Chivers S.J., Danil K., Weller D.W., Hoh E. (2015). Nontargeted biomonitoring of halogenated organic compounds in two ecotypes of bottlenose dolphins (*Tursiops truncatus*) from the Southern California Bight. Environ. Sci. Technol..

[47] Mackintosh S.A., Dodder N.G., Shaul N.J., Aluwihare L.I., Maruya K.A., Chivers S.J., Danil K., Weller D.W., Hoh E. (2016). Newly identified DDT-related compounds accumulating in Southern California bottlenose dolphins. Environ. Sci. Technol..

[48] Cossaboon J.M., Hoh E., Chivers S.J., Weller D.W., Danil K., Maruya K.A., Dodder N.G. (2019). Apex marine predators and ocean health: Proactive screening of halogenated organic contaminants reveals ecosystem indicator species. Chemosphere.

[49] Alonso M.B., Maruya K.A., Dodder N.G., Lailson-Brito J., Azevedo A., Santos-Neto E., Torres J.P., Malm O., Hoh E. (2017). Nontargeted screening of halogenated organic compounds in bottlenose dolphins (*Tursiops truncatus*) from Rio de Janeiro, Brazil. Environ. Sci. Technol..

[50] Vilela R., Huebner M., Vilela C., Vilela G., Pettersen B., Oliveira C., Mendoza L. (2021). The taxonomy of two uncultivated fungal mammalian pathogens is revealed through phylogeny and population genetic analyses. Sci. Rep..

[51] Hall A.J., Hugunin K., Deaville R., Law R.J., Allchin C.R., Jepson P. (2006). The risk of infection from polychlorinated biphenyl exposure in the harbor porpoise (*Phocoena phocoena*): A case-control approach. Environ. Health Perspect..

[52] Van Bressem M.F., Minton G., Collins T., Willson A., Baldwin R., Van Waerebeek K. (2014). Tattoo-like skin disease in the endangered subpopulation of the Humpback Whale, *Megaptera novaeangliae*, in Oman (Cetacea: Balaenopteridae). Zool. Middle East.

[53] Pomilla C., Amaral A.R., Collins T., Minton G., Findlay K., Leslie M.S., Ponnampalam L., Baldwin R., Rosenbaum R. (2014). The World’s Most Isolated and Distinct Whale Population? Humpback Whales of the Arabian Sea. PLoS ONE.

[54] Dakhteh S.M.H., Ranjbar S., Moazeni M., Mohsenian N., Delshab H., Moshiri H., Nabavi S.M.B., Van Waerebeek K. (2017). The Persian Gulf is part of the habitual range of the Arabian Sea Humpback whale population. J. Mar. Biol Oceanogr..

[55] Braun B.A., Marcovitz A., Camp J.G., Jia R., Bejerano G. (2015). Mx1 and Mx2 key antiviral proteins are surprisingly lost in toothed whales. Proc. Natl. Acad. Sci. USA.

[56] Haller S.L., Peng C., McFadden G., Rothenburg S. (2014). Poxviruses and the evolution of host range and virulence. Infect. Genet. Evol..

[57] Chung O., Jung Y.E., Lee K.W., An Y.J., Kim J., Roh Y.R., Bhak J., Park K., Weber J.A., Cheong J. (2022). The Analyses of Cetacean Virus-Responsive Genes Reveal Evolutionary Marks in Mucosal Immunity-Associated Genes. Biochem. Genet..

[58] Smith S.A., Kotwal G.J. (2002). Immune response to poxvirus infections in various animals. Crit. Rev. Microbiol..

[59] Baxby D., Zuckerman A.J., Banatvala J.E., Pattison J.R. (1990). Poxviruses in Principles and Practice of Clinical Virology.

[60] Panchanathan V., Chaudhri G., Karupiah G. (2008). Correlates of protective immunity in poxvirus infection: Where does antibody stand?. Immunol. Cell Biol..

[61] Batley K.C., Sandoval-Castillo J., Kemper C.M., Zanardo N., Tomo I., Beheregaray L.B., Möller L.M. (2021). Whole genomes reveal multiple candidate genes and pathways involved in the immune response of dolphins to a highly infectious virus. Mol. Ecol..

